# 2189. Colistin Heteroresistance in *Enterobacter* due to Base Heterozygosity at Certain *phoPQ* Locations

**DOI:** 10.1093/ofid/ofad500.1811

**Published:** 2023-11-27

**Authors:** Chengcheng Wang, Yu Feng, Zhiyong Zong

**Affiliations:** West China Hospital, Chengdu, Sichuan, China; West China Hospital, Chengdu, Sichuan, China; West China Hospital, Chengdu, Sichuan, China

## Abstract

**Background:**

*Enterobacter* species are major nosocomial pathogens, and the clinical implications and mechanisms of colistin heteroresistance (CHR) in *Enterobacter* remain unclear.

**Methods:**

We used the population analysis profile (PAP) assay to determine the presence of CHR in *Enterobacter.* To examine whether CHR leads to treatment failure, we conducted *in vitro* time-killing assays and *in vivo* assays using murine intra-abdominal infection models. To determine the genetic mechanism for the CHR phenotype, we conducted whole-genome sequencing and ultra-deep second-generation sequencing.

A schematic outline of methods and main results

**Figure:**
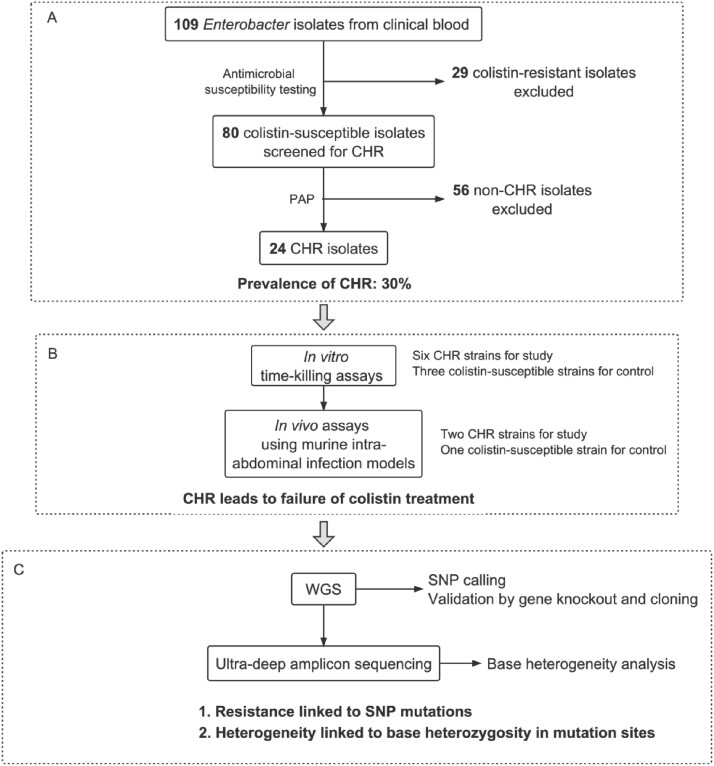

**Results:**

In this study, we found that 30% of *Enterobacter* clinical strains causing bloodstream infections at West China Hospital exhibited CHR, which was associated with treatment failure and fatal infections, as demonstrated by *in vitro* time-kill tests and *in vivo* mouse infection modeling. We identified base alterations in the *phoP-phoQ* gene as the main resistance mechanism in *Enterobacter* CHR and that this heterogeneity originated from colistin selection and base heterozygosity, as detected by ultra-deep second-generation sequencing. We also found that several different resistance subpopulations existed simultaneously in the same strain with different resistance mechanisms.

Time-kill experiments in Enterobacter isolates with CHR or susceptible to colistin

**Figure:**
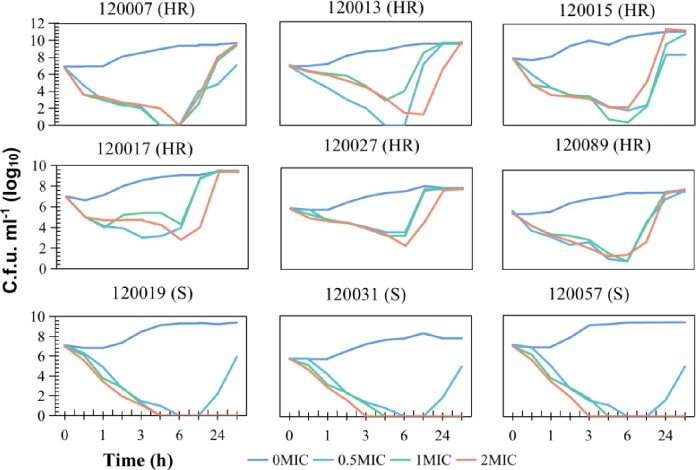

CHR strains lead to in vivo colistin treatment failure

**Figure:**
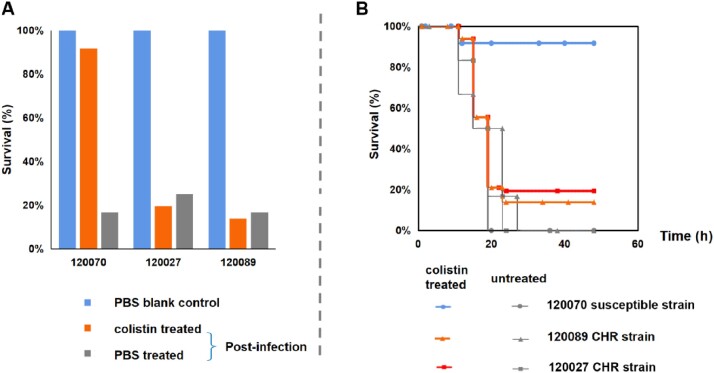

Phenotypic and genomic study of a CHR strain

**Figure:**
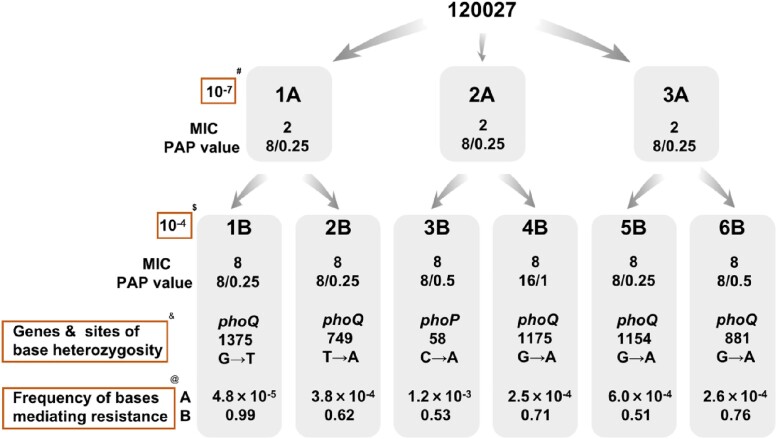

**Conclusion:**

We presented evidence for "genetic heterogeneity" in *Enterobacter* CHR strains, which is consistent with "phenotypic heterogeneity" and genetically explains the unstable and transient HR. This provides important insights and a fresh perspective into the mechanisms and clinical implications of *Enterobacter* CHR and underscores the importance of deep sequencing, prompting the research of high-throughput microbial single-cell sequencing as a method for detecting "genetic heterogeneity."

Frequency of bases that mediate colistin resistance at the same locus of phoP-Q in the parental and resistant strains

**Figure:**
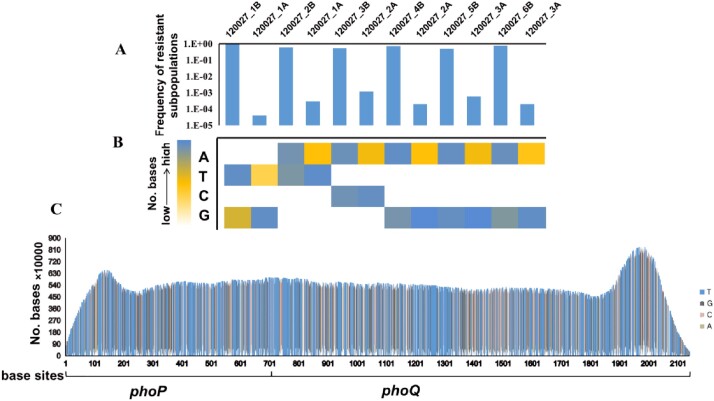

**Disclosures:**

**All Authors**: No reported disclosures

